# Squalestatin alters the intracellular trafficking of a neurotoxic prion peptide

**DOI:** 10.1186/1471-2202-8-99

**Published:** 2007-11-22

**Authors:** Rona Wilson, Clive Bate, Ronald Boshuizen, Alun Williams, James Brewer

**Affiliations:** 1 Division of Immunology, Infection and Inflammation, Western Infirmary, University of Glasgow, G11 6NT, Glasgow; 2 Department of Pathology and Infectious Diseases, Royal Veterinary College, Hawkshead Lane, North Mymms, AL9 7TA, Herts; 3 Pepscan systems B.V, Edelhertweg 15, 8219 PH, Lelystad, The Netherlands; 4 Centre for Biophotonics, Strathclyde Institute for Biomedical Sciences, University of Strathclyde, G4 ONR, Glasgow

## Abstract

**Background:**

Neurotoxic peptides derived from the protease-resistant core of the prion protein are used to model the pathogenesis of prion diseases. The current study characterised the ingestion, internalization and intracellular trafficking of a neurotoxic peptide containing amino acids 105–132 of the murine prion protein (MoPrP105-132) in neuroblastoma cells and primary cortical neurons.

**Results:**

Fluorescence microscopy and cell fractionation techniques showed that MoPrP105-132 co-localised with lipid raft markers (cholera toxin and caveolin-1) and trafficked intracellularly within lipid rafts. This trafficking followed a non-classical endosomal pathway delivering peptide to the Golgi and ER, avoiding classical endosomal trafficking via early endosomes to lysosomes. Fluorescence resonance energy transfer analysis demonstrated close interactions of MoPrP105-132 with cytoplasmic phospholipase A_2 _(cPLA_2_) and cyclo-oxygenase-1 (COX-1), enzymes implicated in the neurotoxicity of prions. Treatment with squalestatin reduced neuronal cholesterol levels and caused the redistribution of MoPrP105-132 out of lipid rafts. In squalestatin-treated cells, MoPrP105-132 was rerouted away from the Golgi/ER into degradative lysosomes. Squalestatin treatment also reduced the association between MoPrP105-132 and cPLA_2_/COX-1.

**Conclusion:**

As the observed shift in peptide trafficking was accompanied by increased cell survival these studies suggest that the neurotoxicity of this PrP peptide is dependent on trafficking to specific organelles where it activates specific signal transduction pathways.

## Background

The Transmissible Spongiform Encephalopathies (TSE)s, otherwise known as prion diseases, are a family of neurodegenerative diseases that include Creutzfeldt-Jakob disease (CJD) in man, Bovine Spongiform Encephalopathy (BSE) in cattle, and scrapie in sheep and goats. A notable feature of these diseases is the accumulation of (PrP^Sc^) [1], a misfolded isoform of the host-encoded prion protein PrP^c ^[2,3]. Neuronal dysfunction and ultimately neuronal death are thought to arise following deposition of fibrils of PrP^Sc ^which accumulate in the brain of infected animals [4-6]. However, it remains unclear if PrP^Sc ^causes neuronal damage itself, or acts via other molecular forms of PrP that have been suggested as causative agents in prion disease [7-9].

The process of neuronal loss can be investigated *in vitro *using highly defined synthetic peptides derived from the protease-resistant core of PrP^Sc^. The majority of neurotoxicity studies have employed a peptide consisting of amino acids 106–126 of the human prion protein (HuPrP106-126) which possesses many of the properties of the PrP^Sc ^isoform, notably a high β-pleated sheet content, fibril formation and toxicity for neurons *in vitro *[7]. A corresponding peptide has been identified from the murine prion sequence (MoPrP105-132), which has also been shown to be neurotoxic [8]. These peptides also encompass the major part of the putative transmembrane form of PrP (^Ctm^PrP) that is thought to be important in prion disease pathogenesis as transgenic mice overexpressing such PrP molecules develop neurological disease, and the accumulation of PrP^res ^is followed closely by an increase in ^Ctm^PrP [10,11]. In the current study we employed labelled MoPrP105-132 to identify organelles involved in the trafficking pathways of neurotoxic peptides. We demonstrate that in neuroblastoma cells, MoPrP105-132 co-localises with cholera toxin subunit B (CTxB), which binds to the ganglioside GM1 [12,13] and caveolin-1 [14,15], markers of specialised microdomains called lipid rafts.

Lipid rafts are highly enriched in cholesterol, sphingolipids and a population of specific membrane proteins [16]. In some cells, lipid rafts contain cholesterol-binding proteins called caveolins that define a subset of lipid raft called caveosomes [17]. Lipid rafts also act as platforms for cell signalling processes [18,19], suggesting that MoPrP105-132 might interact with signalling enzymes. As a close correlation exists between the production of prostaglandins (PG)s and neuronal death in prion disease [20,21], the association between MoPrP105-132 and the enzymes PLA_2 _and COX responsible for the release of arachidonic acid (AA) and the metabolism of AA into PGs respectively, were studied.

There is increasing evidence that cholesterol levels within the brain may affect the progression of some neurodegenerative diseases. Cholesterol depletion *in vitro *has been shown to affect the integrity of lipid rafts [22,23] and reducing cellular cholesterol levels reduces the sensitivity of neurons to prions [23]. In the following studies we have demonstrated that pre-treatment of neurons with squalestatin, a drug that inhibits cholesterol production [24], alters the surface localisation and the intracellular trafficking of the MoPrP105-132 peptide. In addition, pre-treatment with squalestatin significantly reduced the association between MoPrP105-132 and cPLA_2 _or COX-1. Such observations raise the possibility that the neurotoxicity of PrP peptides is dependent on the specific intracellular trafficking pathways of such peptides and the association with signal transduction pathways.

## Results

### MoPrP105-132 is located within lipid rafts

**Figure 1:**
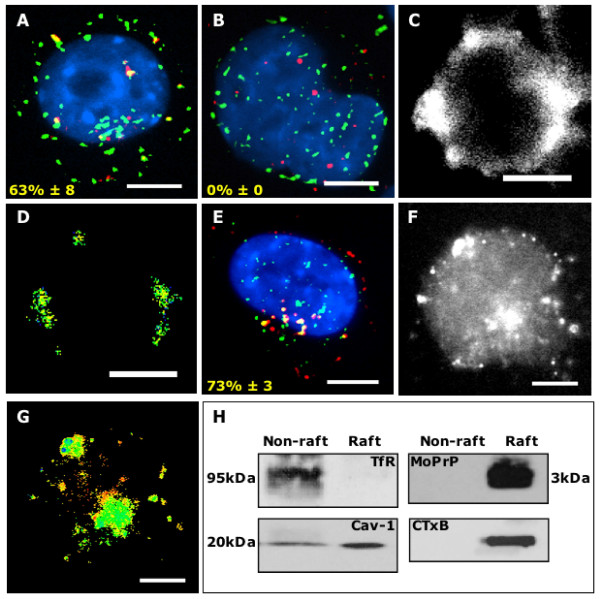
**MoPrP105-132 is found in lipid rafts**. Neuroblastoma cells incubated for 30 minutes with 30 μM rhodamine-labeled MoPrP105-132 and Alexa Fluor 488 labelled CTxB for 30 minutes, the nuclei were revealed using Vectashield with DAPI (blue). (A) Images of MoPrP105-132 (red) and CTxB (green) staining were merged using OpenLab software to show co-localization (yellow). (B) Images of scrambled MoPrP105-132 (red) and CTxB (green) staining showed that there was no co-localization (yellow). (C) FRET analysis showing the raw data between rhodamine-labeled MoPrP105-132 and Alexa Fluor 488 labelled CTxB and (D), the proximity of the donor/acceptor reaction revealed by false-colour intensity. Images of neuroblastoma cells incubated for 30 minutes with 30 μM rhodamine-labeled MoPrP105-132 (red) and stained with FITC-labelled anti-caveolin-1 (green), showing co-localization (yellow). (E) FRET analysis showing the raw data between rhodamine-labeled MoPrP105-132 and FITC-labelled CTxB and (F), the proximity of the donor/acceptor reaction revealed by false-colour intensity. Scale bars, 5 μm. (H), Western-blot analysis of triton X-100 insoluble fractions (lipid rafts) and soluble fractions (non-raft) isolated from neuroblastoma cells incubated with 30 μM MoPrP105-132-FITC for 30 minutes, and probed for MoPrP105-132, caveolin-1, TfR and GM1 (CTxB).

Initial studies demonstrated that to achieve internalisation and detection of labelled MoPrP105-132 in greater than 50% of the NB4 cells required at least 30 – 90 minutes incubation at 37°C (data not shown). Following this incubation period, 63% ± 8 of rhodamine-labelled MoPrP105-132 co-localised with Alexa Fluor 488 labelled CTxB, which binds to ganglioside-GM1 and identifies lipid rafts [13-15] (Figure 1A). In contrast, no co-localisation between scrambled MoPrP105-132 and CTxB was detected (Figure 1B), indicating that the localisation of MoPrP105-132 in lipid rafts was dependent on the primary sequence of the peptide. To confirm these findings on non-transformed cells, further experiments were conducted using primary cortical neurons; comparable results were obtained (see additional files 1A &1B). A FRET signal generated between acceptor-conjugated MoPrP105-132 and donor conjugated CTxB indicated that the two molecules were in close association, approximately 10–100 Å (Figures 1C, 1D). In contrast, no FRET signal could be detected between scrambled MoPrP105-132 and CTxB (data not shown). To determine whether caveolae played a role in the trafficking of MoPrP105-132, neuroblastoma cells were incubated with rhodamine-labelled MoPrP105-132 for 30 minutes, fixed and probed with FITC labelled anti-caveolin-1. We found 73% ± 3 of MoPrP105-132 co-localised with caveolin-1 (Fig 1E). Furthermore, a FRET signal between MoPrP105-132 and caveolin-1 was also detected confirming the close association of these molecules (Figures 1F &1G). The fluorescence microscopy studies were complemented by lipid rafts isolation studies on MoPrP105-132 treated neuroblastoma cells. Following incubation for 30 minutes, MoPrP105-132 was found in a TfR negative, caveolin-1 and CTxB positive fraction (Figure 1H). These results confirm that MoPrP105-132 can be found in lipid rafts and that caveolin-1 was also present in the lipid rafts that were isolated.

### MoPrP105-132 traffics via a non-classical endocytic pathway

**Figure 2:**
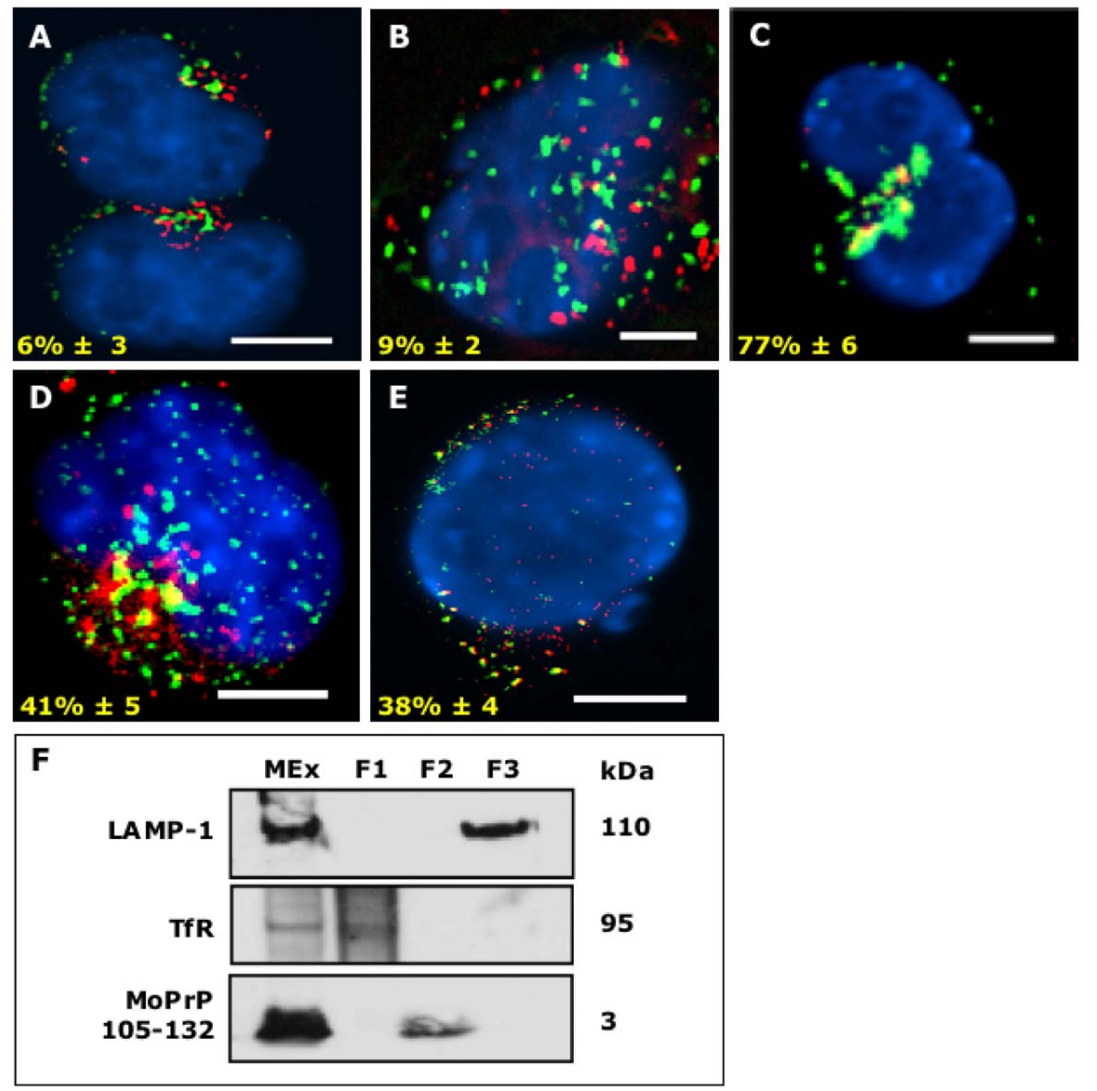
**MoPrP105-132 avoids early endosomes and lysosomes**. Neuroblastoma incubated with biotinylated MoPrP105-132 at 37°C, then fixed and stained with Texas Red-streptavidin (red) and TfR-FITC (green) or LAMP-1-FITC (green). Nuclei were revealed using Vectashield with DAPI (blue). (A) Co-localisation (yellow) was not observed between MoPrP105-132 and TfR or (B) between MoPrP105-132 and LAMP-1. (C) Co-localisation of biotinylated scrambled MoPrP105-132 (red) with LAMP-1 (green) was evident. Neuroblastoma cells were incubated with biotinylated MoPrP105-132 (red) for 90 minutes, then fixed and stained with GM130 (green) or Grp78 (green). (D) Co-localisation was observed between MoPrP105-132 and GM130 and (E) between MoPrP105-132 and Grp78. Scale bars, 5 μm. (F) Separation of endosomal compartments. Neuroblastoma cells were pulsed with iron dextran beads, before incubation with MoPrP105-132-FITC for 1 hour. Lysosomes (F3) were extracted from whole microsome extracts (MEx) using a magnetic column and early endosomes (F1) and intermediate fraction (F2) isolated using a density gradient. Western blot analysis revealed MoPrP105-132 was present in TfR negative and LAMP-1 negative fractions (F2).

The intracellular trafficking pathway of MoPrP105-132 was further investigated by incubating biotin-conjugated MoPrP105-132 for 1 hour at 37°C before fixation. Fluorescence microscopy showed that only 6% ± 3 of MoPrP105-132 localised in TfR positive early endosomes (Figure 2A) and only 9% ± 2 of MoPrP105-132 co-localised within LAMP-1 positive lysosomes (Figure 2B). In contrast, 77% ± 6 of scrambled MoPrP105-132 was associated with LAMP-1 positive lysosomes (Figure 2C), confirming the sequence dependence of MoPrP105-132 localisation. Comparable results were obtained in primary cortical neurons (see additional files 2A, 2B). After 90 minutes at 37°C, approximately 41% ± 5 of MoPrP105-132 co-localised with GM130, a marker for cis-Golgi (Fig 2D) and 38% ± 4 with Grp78, which identifies the ER (Fig 2E). These findings are consistent with previous reports that that molecules internalised in lipid rafts traffic to the Golgi/ER [25]. The microscopy studies were complemented by an endosomal fractionation technique, as previously described [26]. In neuroblastoma cells incubated with MoPrP105-132 for 1 hour at 37°C, MoPrP105-132 was detected in the whole microsomal extract and the cell fraction known to be enriched for Golgi and ER compartments [27], but was not found in either TfR positive or LAMP-1 positive fractions (Figure 2F).

### Squalestatin treatment alters MoPrP105-132 trafficking

**Figure 3:**
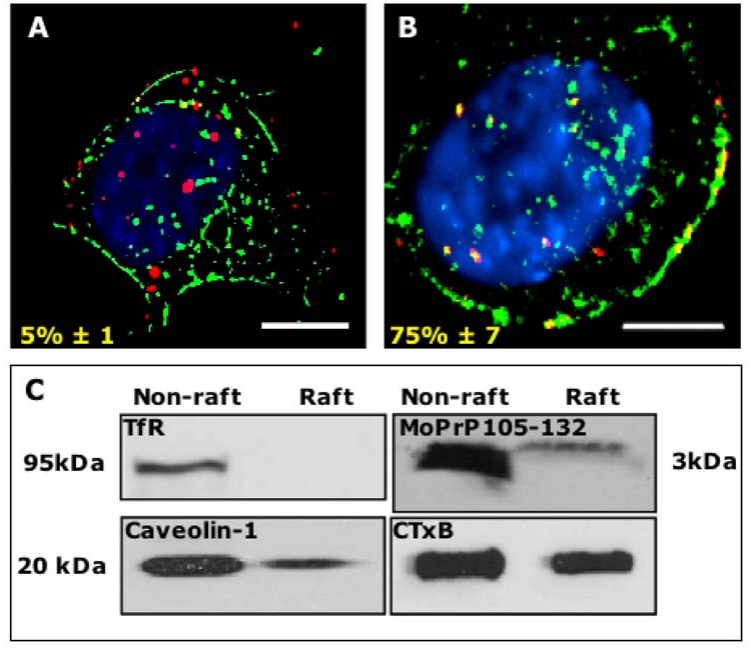
**Squalestatin reduces the distribution of MoPrP105-132 and CTxB in neuroblastoma cells**. Neuroblastoma cells were incubated with 1 μM squalestatin for 24 hours and subsequently incubated with MoPrP105-132-rhodamine (red) and CTxB-Alexa Fluor 488 (green), nuclei were revealed using Vectashield with DAPI (blue). (A) Lack of co-localisation was apparent between MoPrP105-132 and CTxB in squalestatin treated cells compared with (B) untreated cells. Scale bar, 5 μm. (C) Isolation of lipid raft/non-raft membranes from neuroblastoma cells treated with 1 μM squalestatin for 24 hours. In squalestatin-treated cells the majority of MoPrP105-132 was present in the non-raft fraction.

Previous studies have shown that depletion of cholesterol in neurones protects against the neurotoxicity induced by prion peptides [23], including MoPrP105-132 (see additional data 3). To determine whether squalestatin was simply altering the quantity of MoPrP105-132 ingested, squalestatin treated or untreated neuroblastoma cells were incubated with 30 μM MoPrP105-132 conjugated to FITC. The levels of cell associated fluorescence in each condition was determined after 30 minutes incubation by FACS analysis of cells and expressed as arbitrary units of mean fluorescence intensity. There was no significant difference between the mean fluorescence intensity for untreated and squalestatin treated cells (23.2 ± 2 compared to 25.3 ± 4, n = 6, P > 0.05). As a number of studies have demonstrated that lipid rafts involved in internalisation and trafficking are cholesterol sensitive, the effect of squalestatin on the intracellular trafficking of MoPrP105-132 was investigated. Following pre-treatment with squalestatin, neuroblastoma cells were incubated with CTxB and MoPrP105-132 for 30 minutes at 37°C. Fluorescence microscopy revealed that in squalestatin-treated cells only 5% ± 1 of the MoPrP105-132 co-localised with CTxB (Figure 3A), whereas 75% ± 7 of MoPrP105-132 co-localised with CTxB in untreated neuroblastoma cells (Figure 3B). When lipid rafts were isolated from neuroblastoma cells that had been pre-treated with squalestatin, most of the MoPrP105-132 was detected in the non-raft fraction, while CTxB and caveolin-1 were present in both raft and non-raft fractions (Figure 3C). Such findings suggest that the localisation of MoPrP105-132 to lipid rafts is sensitive to cholesterol depletion.

**Figure 4:**
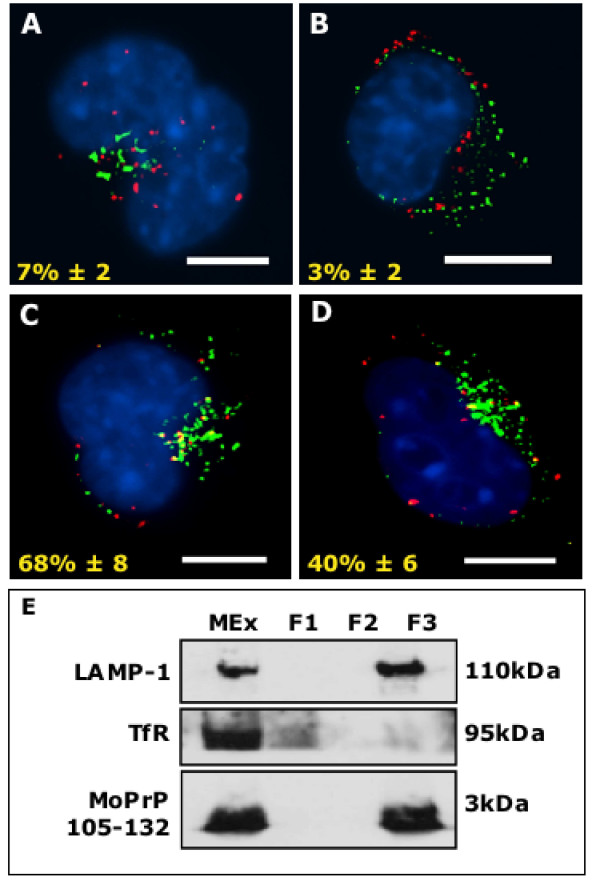
**Squalestatin reroutes MoPrP105-132 into lysosomes**. Neuroblastoma cells were incubated with 1 μM squalestatin for 24 hours before incubation with 30 μM MoPrP105-132-biotin for 90 minutes, then fixed and stained with Texas Red-streptavidin (red) and with anti-GM130, anti-Grp78, anti-TfR-FITC or anti-LAMP-1-FITC (all green) (A) Co-localisation between MoPrP105-132 and GM130, or (B) between MoPrP105-132 and Grp78. (C) In squalestatin treated cells co-localisation was observed between MoPrP105-132 and TfR and (D) between MoPrP105-132 and LAMP-1. Scale bars, 5 μm. (E) In squalestatin-treated cells, following fractionation of whole microsomal extracts (MEx) using iron dextran and density gradient centrifugation, MoPrP105-132 was detected in the lysosome fraction (F3).

Further analysis of the trafficking of MoPrP105-132 in squalestatin-treated neuroblastoma cells showed that only 7% ± 2 of MoPrP105-132 co-localised with the Golgi (Figure 4A) and 3% ± 2 of MoPrP105-132 co-localised with the ER (Figure 4B), suggesting that in these cells MoPrP105-132 does not undergo retrograde trafficking. We found that 68% ± 8 of MoPrP105-132 co-localised with early endosomes (Figure 4C) and 40% ± 6 of MoPrP105-132 co-localised with lysosomes (Figure 4D). These observations suggest that in squalestatin-treated cells the MoPrP105-132 peptide is diverted away from retrograde transport to the Golgi/ER and directed into the classical endosome/lysosomal degradative pathway. A summary of the differences in the trafficking pathways of MoPrP105-132 in untreated and squalestatin-treated neuroblastoma cells is presented as Table 1. The re-routing of MoPrP105-132 to the classical endocytic pathway in squalestatin-treated cells was confirmed by analysis of endosomal fractions. In endosomal fractions isolated from squalestatin-treated neuroblastoma cells pulsed with MoPrP105-132 for 1 hour, MoPrP105-132 was detected in the LAMP-1 positive, TfR negative, lysosomal fraction (Figure 4E).

**Table 1:** Squalestatin alters the trafficking of MoPrP105-132 in neuroblastoma cells. The percentage of MoPrP105-132 that co-localised with cellular markers as shown in untreated neuroblastoma cells and cells treated for 24 hours with 1 μM squalestatin. Values shown are mean percent of peptide that co-localises with markers as shown, ± SD, which was calculated after counting a minimum of 3 cells.

	**% Co-localisation with MoPrP105-132**	
	
	Untreated	Squalestatin-treated
CTxB	63% ± 8	5% ± 1
Caveolin-1	73% ± 3	9% ± 3
GM130	41% ± 5	7% ± 2
Grp 78	38% ± 4	3% ± 2
TfR	6% ± 3	68% ± 8
LAMP-1	9% ± 2	40% ± 6

### MoPrP105-132 associates with cPLA_2 _and COX-1

**Figure 5:**
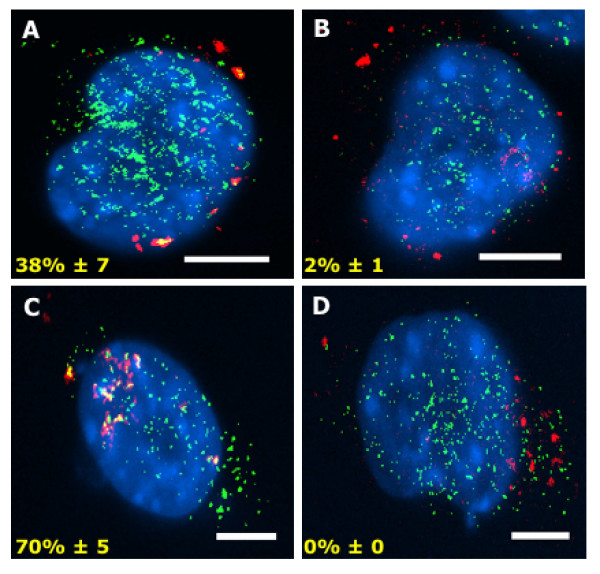
**MoPrP105-132 associates with cPLA_2 _and COX-1**. Neuroblastoma cells were incubated with 30 μM MoPrP105-132-biotin for 5 minutes or 20 minutes at 37°C then fixed and stained with Texas Red-streptavidin (red), anti-cPLA_2_-FITC (green) or anti-COX-1-FITC (green). Nuclei were revealed using Vectashield with DAPI (blue). (A) Co-localisation (yellow) between MoPrP105-132 (red) and cPLA_2 _(green) after 5 minutes, but not after 20 minutes (B). (C) Co-localisation (yellow) between MoPrP105-132 (red) and anti-COX-1-FITC (green) after 5 minutes, but not after 20 minutes (D). Scale bars, 5 μm.

Since a close association exists between PG's and prion-induced neurotoxicity [28-31], the relationship between MoPrP105-13 and enzymes involved in the production of PGs was examined. Neuroblastoma cells were incubated with MoPrP105-132 for either 5 minutes or 20 minutes at 37°C, fixed, and stained with antibodies to either cPLA_2 _or COX-1. After 5 minutes 38% ± 7 of MoPrP105-132 co-localised with cPLA_2 _(Figure 5A) and 70% ± 5 of MoPrP105-132 co-localised with COX-1 (Figure 5C). After 20 minutes only 2% ± 1 of MoPrP105-132 co-localised with cPLA_2 _(Figure 5B) and no co-localisation was observed between MoPrP105-132 and COX-1 (Figure 5D), indicating that the association between MoPrP105-132 and cPLA_2 _or COX-1 was short lived.

### Squalestatin treatment reduces the association between MoPrP105-132 and cPLA_2_/COX-1

**Figure 6:**
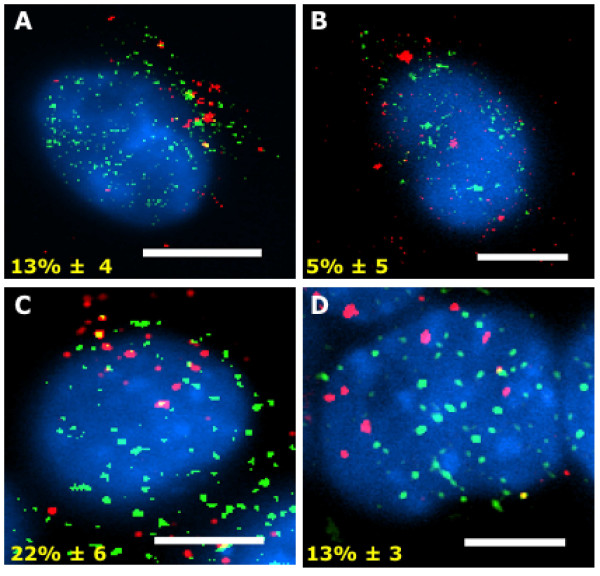
**Squalestatin reduces the association between MoPrP105-132 and cPLA_2 _and COX-1**. Neuroblastoma cells were pre-treated with either 1 μM squalestatin or 2 μM Hexa-PAF prior to incubation with 30 μM MoPrP105-132-biotin for 5 minutes at 37°C, fixed and stained with Texas Red-streptavidin (red) anti-cPLA_2_-FITC or anti-COX-1-FITC (green). Nuclei were revealed using Vectashield with DAPI (blue). Images show the extent of co-localisation (yellow) between MoPrP105-132 (red) and cPLA_2 _(green) (A) or between MoPrP105-132 (red) and COX-1 (green) (B) in squalestatin-treated cells. Similarly, images show the extent of co-localisation (yellow) between MoPrP105-132 (red) and cPLA_2 _(green) (C) or between MoPrP105-132 (red) and COX-1 (green) (D) in Hexa-PAF-treated cells. Scale bars, 5 μm.

Since treatment with squalestatin protected neurons against prions [23] the effects of squalestatin on the co-localisation between MoPrP105-132 and cPLA_2 _was examined 5 minutes after the addition of peptide. Pre-treatment with 1 μM squalestatin significantly reduced the association between MoPrP105-132 and cPLA_2 _(13% ± 4 compared with 38% ± 7 in untreated cells) (Figure 6A). Similarly, pre-treatment with squalestatin reduced the association between MoPrP105-132 and COX-1 (5% ± 5 compared to 70% ± 5 in untreated cells) (Figure 6B). Neurons treated with PAF antagonists are also resistant to the otherwise toxic effects of prions [23] and pre-treatment with 2 μM of a PAF antagonist (Hexa-PAF) reduced the association between MoPrP105-132 and cPLA_2 _(22% ± 6 v 38% ± 7 in untreated cells) (Figure 6C). Treatment with Hexa-PAF also reduced the association between MoPrP105-132 and COX-1 (13% ± 3 compared to 70% ± 5 in untreated cells) (Figure 6D). A summary of the differences in co-localisation between MoPrP105-132 and the signalling enzymes in untreated and squalestatin-treated neuroblastoma cells is presented as Table 2.

**Table 2:** Squalestatin reduces co-localisation between MoPrP105-132 and cPLA_2_/COX-1. The percentage of MoPrP105-132 that co-localised with the signalling enzymes cPLA_2 _or COX-1 in untreated neuroblastoma cells and cells treated for 24 hours with either 1 μM squalestatin or 2 Hexa-PAF, 5 minutes after the addition of peptide. Values shown are mean ± SD, calculated after counting a minimum of 3 cells.

**Co-localisation with MoPrP105-132**	**Drug Treatments**		
	
	Untreated	Squalestatin	Hexa-PAF
cPLA_2_	38% ± 7	13% ± 4	22% ± 6
COX-1	70% ± 5	5% ± 5	13% ± 3

## Discussion

Although neuronal dysfunction and ultimately neuronal loss are key features of prion diseases, the molecular mechanisms that that result in neuronal damage remain poorly understood. Synthetic peptides have been extensively used to model the process of neuronal degeneration *in vitro *and in the present study we analysed the trafficking of MoPrP105-132 in neuroblastoma cells and primary cortical neurones. Most of the MoPrP105-132 molecules co-localised with ganglioside GM1, a marker of lipid rafts identified by CTxB, and with caveolin-1, a marker of a lipid raft subset called caveolae. FRET analysis, a technique with spatial resolution beyond the limits of conventional fluorescence imaging, demonstrated that MoPrP105-132 was intimately associated with both GM1 and caveolin-1 (minimal spacing of 10–100 Å required to observe FRET signals). Separation of lipid rafts from cell membranes by detergent extraction confirmed the microscopy data (MoPrP105-132 was detected only in the GM1 and caveolin-1 positive fraction). It not clear why MoPrP105-132 localises to lipid rafts, one possibility is that lipid rafts contain a high percentage of saturated fatty acids, which provides a hydrophobic environment that stabilises the peptide within the membrane. Another possibility is that many of the putative receptors for prions [32] are found within lipid rafts, thus, peptide-receptor interactions may be responsible for targeting MoPrP105-132 to these domains.

The trafficking of molecules within cells is partly dependent on the mechanism of internalisation, thus, molecules that are ingested via clathrin-dependent endocytosis traffic via endosomes into degradative lysosomes [33]. In contrast, agents targeted to lipid rafts or caveolae are internalised by clathrin-independent endocytosis and traffic into different cellular compartments. MoPrP105-132 trafficked into both the Golgi and ER (co-localised with the markers GM130 and Grp78); consistent with reports that the cargo of caveolae is directed into the Golgi [14,15,34]. These results are consistent with MoPrP105-132 trafficking via the non-classical endocytic pathway, recently defined through the trafficking of GM1 positive lipid rafts [12]. Only a small percentage of MoPrP105-132 was associated with either TfR or LAMP-1, indicating that it was rarely found in early endosomes or lysosomes. Microscopy data was supported by organelle fractionation studies in which endosomes involved in the classical endocytic pathways were fractionated. MoPrP105-132 was not found in fractions containing either TfR or LAMP-1, but was found in an intermediate density fraction known to contain Golgi/ER [27]. Taken together these results indicate that MoPrP105-132 does not traffic down the classical endocytic route and avoids the lysosomes. We speculate that the avoidance of proteolytic lysosomes may facilitate the build up of neurotoxic peptides during disease progression. The localisation of MoPrP105-132 to lipid rafts depends on the sequence of amino acids as a control peptide made up of the same amino acids synthesized in a scrambled sequence did not localise within lipid rafts. Subsequently, the scrambled MoPrP105-132 control peptide trafficked to endosomes and lysosomes, emphasising the sequence dependent nature of the intracellular trafficking of the peptide. Although most of the studies presented are on neuroblastoma cells we found that MoPrP105-132 trafficked in a similar manner in non-transformed primary cortical neurones. The sequence specificity of this effect would argue against MoPrP non-specifically associating with lipid rafts through for example, hydrophobicity, as the composition of the scrambled peptide was the same as MoPrP105-132.

Previous studies demonstrated that neurons treated with the cholesterol synthesis inhibitor squalestatin, a drug that inhibits squalene synthase and reduces cholesterol production without affecting the production of isoprenoid precursors [24], were protected against the otherwise toxic effects of prions [23]. Since the formation of lipid rafts and lipid raft-dependent uptake is sensitive to cholesterol depletion [35], we examined the effects of squalestatin-treatment on the trafficking of MoPrP105-132. One possible explanation for the neuroprotective effects of squalestatin, that it prevents ingestion of MoPrP105-132, was excluded by studies that showed that there was no significant difference in the amounts of peptide taken up by untreated and treated cells. However, we did detect a difference in the trafficking of MoPrP105-132 between treated and untreated cells. Squalestatin-treatment of neuroblastoma cells greatly reduced the amounts of MoPrP105-132 that co-localised with either CTxB or with caveolin-1 indicating the most of the peptide was no longer present in lipid rafts. This observation was confirmed by Triton X-100 extractions which showed that most of the MoPrP105-132 peptide was now in the non-raft fraction. Fluorescence microscopy and cell fractionation studies both suggested that in squalestatin-treated cells MoPrP105-132 trafficked down a classical endocytic pathway into lysosomes, avoiding the Golgi/ER. These results are consistent with reports that cholesterol depletion results in the loss of lipid raft functions, including raft-dependent endocytosis and subsequent intracellular trafficking [36].

The neurotoxicity of prion-derived peptides depends on the activation of specific signalling pathways including PLA_2 _and the COX enzymes that convert AA into PGs [28-31]. Furthermore, prion-induced injury is associated with increased levels of PGE_2 _[20,21]. Selective inhibitors indicated the importance of the COX-1 isoform in prion-mediated neurotoxicity [28]. In untreated neuroblastoma cells a significant proportion of MoPrP105-132 co-localised with cPLA_2 _and COX-1, this association was short lived (seen at 5 minutes after the addition of peptide but not after 20 minutes). The brief association observed could be a result of peptide rapidly transiting the COX-1/cPLA2 containing compartment, or alternatively localisation of these enzymes is known to be transient and dependent on the activation state of the cell [37]. These observations suggest that the association of MoPrP105-132 with cPLA_2 _and COX-1 activates these enzymes leading to the production of toxic second messengers. Pre-treatment of neuroblastoma cells with squalestatin reduced the co-localisation between MoPrP105-132 and cPLA_2 _or COX-1, and reduced prion-induced PGE_2 _production [23]. Taken together these observations suggest that in normal neuroblastoma cells MoPrP105-132 accumulates in lipid rafts where they activate the PLA_2_/COX pathway resulting in the production of neurotoxins including PGE_2 _[28]. Thus, the neuroprotective effect of squalestatin treatment may simply be by the dispersal of lipid rafts, which prevent the formation of MoPrP105-132/PLA_2_/COX-1 complexes and reduce the production of neurotoxins. Similar effects where found in neuroblastoma cells treated with the PAF antagonist (Hexa-PAF), which protects neurones against prion-mediated neuronal damage [30]. Pre-treatment of neuroblastoma cells with the PAF antagonist reduced the co-localisation between MoPrP105-132 and cPLA_2_, and significantly between MoPrP105-132 and COX-1. This observation is consistent with reports that PAF antagonists reduce PGE_2 _production in prion peptide treated neuroblastoma cells [38].

## Conclusion

In conclusion, our data demonstrate that in normal neuroblastoma cells the majority of MoPrP105-132 is found in lipid rafts and traffics via a non-classical endocytic, recycling pathway. This pathway involves internalisation via lipid rafts and trafficking into the Golgi and ER. During this process MoPrP105-132 co-localises with the signalling enzymes, cPLA_2 _and COX-1, enzymes that are involved in the production of prostaglandins, bioactive lipids closely associated with neuronal death in prion diseases. Treatment of neuroblastoma cells with squalestatin was shown to prevent peptide-induced neurotoxicity but did not prevent internalisation. However squalestatin treatment was associated with altered localisation and trafficking of MoPrP105-132 in neuroblastoma cells. In these cells MoPrP105-132 is not found in lipid rafts, did not associate with cPLA_2 _and COX-1, and trafficked into lysosomes. We speculate that the effect of squalestatin in negating prion peptide induced neurotoxicity may result from altered trafficking of MoPrP105-132 thus preventing interactions with specific signalling pathways. This hypothesis is strengthened by the observation that a second neuroprotective drug, Hexa-PAF, also prevented peptide association with cPLA_2 _and COX-1. As altering the trafficking of prion-derived peptides appears to be associated with protection against neurotoxicity, strategies to manipulate intracellular trafficking pathways could lead to novel therapeutic approaches in prion disease.

## Methods

### Cell Lines

The murine neuroblastoma NB4-1A3 cell line (European Collection of Cell Cultures) was maintained in RPMI-1640 (Invitrogen, Paisley, UK) supplemented with 2 mM glutamine and 5% foetal calf serum (FCS). Cells were dispensed onto sterilised 13 mm glass coverslips pre-coated with poly-D-lysine (50 μg/ml) at 3 × 10^5 ^cells per well (24 well plates) and left to adhere overnight before further use.

### Primary neuronal cultures

Primary cortical neurons were prepared from embryonic day 15.5 mice as previously described [39] and plated onto sterilised 13 mm glass coverslips pre-coated with poly-D-lysine (50 μg/ml) at 2 × 10^6 ^cells per well (24 well plates) [39]. After 2 hours, media was changed to neurobasal medium (NBM) containing B27 components (Invitrogen), 2 mM glutamine and 5 μM cytosine arabinoside (Sigma, Dorset, UK) to prevent the proliferation of astroglia. Neuronal cultures were used 7 days after plating.

### PrP Peptides

Synthetic PrP peptides were derived from the sequence of murine prion protein (MoPrP105-132; KTNLKHVAGAAAAGAVVGGLGGYMLGSA) [8] as was a control peptide containing the same amino acids in a scrambled order (scrambled MoPrP105-132; NGAGKAGMVGLYGAHGATAKVSLVGALA). Peptides were synthesized by solid-phase chemistry and purified by reverse-phase HPLC (gift from Dr. J. Langeveld, ID-Lelystad/Pepscan Systems, The Netherlands). To enable detection of the peptides, they were labelled with rhodamine, fluorescein or biotin.

### Fluorescence microscopy

NB4 1A3 neuroblastoma cells were incubated with 30 μM rhodamine or biotinylated MoPrP105-132 or scrambled peptide conjugated to rhodamine or biotin for 20 to 90 minutes at 37°C. Biotinylated MoPrP105-132 was detected using streptavidin Texas Red (Vector Laboratories, Peterborough, UK). Lipid rafts were identified using 1 μg/ml CTxB conjugated to Alexa Fluor 488 (Molecular Probes, The Netherlands) for 30 minutes at 37°C. FITC-dextran (70 kDa; Molecular Probes) was used as a marker for fluid-phase uptake. For intracellular staining, cells were fixed with 1% paraformaldehyde, Following incubation, cells attached to coverslips were washed once in PBS and fixed in 1% paraformaldehyde. Lipid rafts were identified using 1 μg/ml CTxB conjugated to Alexa Fluor 488 (Molecular Probes, The Netherlands) for 30 minutes at 37°C. For intracellular staining, cells were permeabilised (PBS containing 2% FCS, 2 mM EDTA, 0.1% w/v saponin) and incubated in blocking buffer (PBS containing 1% FCS) to prevent non-specific binding. Biotinylated MoPrP105-132 was detected using streptavidin Texas Red (Vector Laboratories, Peterborough, UK). Early endosomes and lysosomes were stained using antibodies specific for the transferrin receptor (TfR-FITC; BD Pharmingen, Oxford, UK) and lysosomal associated membrane protein-1 (LAMP-1-FITC; BD Pharmingen) respectively. The Golgi was stained using GM130 (Santa Cruz Biotechnology, CA, USA), a marker for the Golgi, and visualised using anti-rabbit FITC (Vector Laboratories). In addition, the ER was stained using anti-Grp78 (Stressgen Biotechnology, San Diego, USA) and visualised using anti-rabbit FITC (Vector Laboratories). Anti-COX-1-FITC (BD Biosciences) and anti-cPLA_2_-FITC (Santa Cruz Biotechnology, California, USA) were used for investigating signalling enzymes. Vectashieid containing DAPI (Vector Laboratories) was used to stain cell nuclei. Coverslips were mounted onto glass slides (Speci-Microsystems Ltd, Surrey, UK) and fluorescence microscopy was performed with an Axiovert S-100 Zeiss microscope using a 63× oil immersion lens. Serial images in z were captured using a Hamamatsu Orca LCD camera and unprocessed images (additional file 4) were deconvolved using OpenLab software (Improvision, Coventry, UK). Images shown are representative of at least 10 fields of view, with a cell density of typically 5 – 10 cells per field. The degree of co-localisation in fluorescent deconvolved images was evaluated from the number of co-localised pixels within a given cell for a minimum of 3 representative cells, as determined by OpenLab software. For drug treatment, cells were pre-treated under the following conditions prior to the addition of prion peptides: 1 μM squalestatin (GlaxoSmithKline, Herts, UK) or 2 μM 1-*O*-alkyl-2-acetyl-*sn*-glycerol-3-phospho-(N,N,N-trimethyl)-hexanolamine (Hexa-PAF) (Novabiochem, Nottingham, UK) overnight at 37°C before use.

### FRET

NB4 1A3 cells were incubated with 30 μM MoPrP105-132 conjugated to rhodamine and either CTxB conjugated to Alexa Fluor 488, or FITC-labelled caveolin-1 for 30 minutes at 37°C. Fluorescence microscopy was performed with an Olympus BX50 fluorescence microscope equipped with appropriate excitation and emission filter sets. Images representing donor fluorescence, acceptor fluorescence (rhodamine) and FRET signal were taken. Donor and acceptor bleed through values were calculated and the FRET signal quantified and FRET images generated using OpenLab FRET module software [40].

### Lipid raft isolation

Lipid raft isolation was performed as previously described [41]. Briefly, NB4 1A3 cells were incubated with 30 μM MoPrP105-132-FITC for 30 minutes, before being lysed with distilled H_2_O and centrifuged (10 minutes at 1,000 × *g*). Pellets were suspended in 1% Triton-X 100 in PBS plus 5 mM phenylmethyl sulfonyl fluoride (PMSF) and incubated for 30 minutes on ice. After centrifugation (10 minutes at 14,000 × *g*), the supernatant was kept as the non-lipid raft fraction. Pellets were suspended in extraction buffer (10 mM Tris-HCL, 10 mM NaCl, 10 mM EDTA, 0.5% Nonidet P-40 and 0.5% Sodium Deoxycholate) and centrifuged (10 minutes at 14,000 × *g*). The supernatant obtained contained the lipid raft fraction. Using western blot analysis, fractions were transferred onto nitrocellulose and probed for MoPrP105-132 using anti-FITC horseradish peroxidase (HRP;Molecular Probes). Fractions were probed using anti-TfR-FITC and anti-Caveolin-1-FITC (Santa Cruz Biotechnology) and detected using anti-FITC HRP. Fractions were visualised using enhanced chemiluminescence (ECL). Using slot blot analysis, ganglioside-1 (GM1) was detected using CTxB-FITC (Sigma) followed by anti-FITC HRP. For drug treatment, NB4 1A3 cells were pre-treated with squalestatin as described above.

### Cell homogenisation and fractionation

NBA 1A3 cells were fractionated using a combination of magnetic isolation of lysosomes [26] and gradient centrifugation [42,43]. Cells were incubated with iron dextran, produced from 40 kDa dextran as previously described [44] for 1 hour at 37°C. Media containing iron dextran was removed and replaced with fresh medium and incubated overnight. After incubation with 30 μM MoPrP105-132-FITC for 1 hour at 37°C, NB4 1A3 cells were scraped into cold (4°C) homogenisation buffer (0.5 mM EGTA, 0.5 mM EDTA, 20 mM HEPES, 0.05% gelatin and 250 mM sucrose). Cells were centrifuged (250 × *g *for 8 minutes) and suspended in 1 ml homogenisation buffer and then lysed by repeat passage through a 25 G syringe. Nuclei and large fragments were removed by low speed centrifugation (110 × *g *for 6 minutes) and the resultant supernatant was then applied to a MACS column (Miltenyi Biotec, Surrey, UK) and allowed to run through the column by gravity to give a non-lysosomal fraction. The column was then washed with homogenisation buffer before being removed from the magnet and the remaining homogenate was eluted, giving a lysosomal fraction [F3]. Using a density gradient technique [26], the non-lysosomal fraction was layered on top of a two step 17% (w/w) Percoll (Amersham Biosciences, UK), 64% (w/w) sucrose cushion. After ultracentrifugation (1 hour at 80,000 × *g*) using a Beckman Type 70Ti fixed angle rotor, bands containing endosomal organelles [F1] and Golgi and ER [F2] were removed and concentrated by centrifugation (100,000 × *g *for 30 minutes). The protein content of these fractions were standardised following protein assay (Pierce MicroBCA kit; Pierce, Cheshire, UK). Gel electrophoresis of the endosomal fractions was performed using precast 4–12% Bis-Tris gradient gels with 2-(N-morpholino)ethanesulfonate (MES) running buffer (NuPAGE; Invitrogen) and proteins were transferred onto nitrocellulose, using western blot analysis and probed for MoPrP105-132 using anti-FITC HRP. Secondary antibody anti-FITC HRP was used to detect TfR-FITC and LAMP-1-FITC and fractions were visualised using enhanced chemiluminescence ECL. For drug treatment, NB4 1A3 cells were pre-treated with squalestatin as described above.

### Flow Cytometry

NB4 1A3 cells were incubated with FITC-conjugated MoPrP105-132 for 30 minutes at 37°C. Cells were then centrifuged and fixed with 1% paraformaldehyde for 10 minutes, washed and suspended in FACS flow (BD, Pharmingen). Cell associated fluorescence was determined by FACS analysis (FACSCalibur; Becton Dickinson), and expressed as arbitrary fluorescence units (mean fluorescence intensity) using Cell Quest software. Flow cytometry was also performed on MoPrP105-132-FITC treated, NB4 1A3 cells that had been pre-treated with either squalestatin as described above.

### Statistical Analysis

Treatment effects were compared using one and two way analysis of variance techniques as appropriate. For all statistical tests, significance was set at the 1% level. Percentage co-localisation in fluorescent deconvolved images was determined by counting the number of co-localised pixels against the number of pixels that did not co-localise, using OpenLab software. Percentage of co-localisation was presented as% ± standard deviation (SD), which was calculated after counting a minimum of 3 cells.

## List of abbreviations

PrP^Sc^, Scrapie isoform of PrP; PrP^C^, Cellular isoform of prion protein; ^Ctm^PrP, transmembrane form of PrP; MoPrP, murine prion protein; GPI, glycosylphophatidylinositol; CTxB, cholera toxin subunit B; ER, Endoplasmic Reticulum; PG, prostaglandin; PLA_2_, phospholipase A_2_; AA, Arachidonic acid; COX, cyclo-oxygenase; PAF, platelet activating factor; cPLA_2_, cytoplasmic phospholipase A_2_; NBM, Neurobasal medium; FCS, Fetal calf serum; TfR, transferrin receptor; Lamp-1, lysosomal associated membrane protein-1; FRET, fluorescence resonance energy transfer; PMSF, phenylmethyl sulfonyl fluoride; HRP, horseradish peroxidase; ECL, enhanced chemiluminenscence; GM1, ganglioside-1.

## Authors' contributions

RKW carried out the fluorescence microscopy, fractionation studies and statistical described and drafted the manuscript. RB produced the labelled peptides for the study. JMB, RB, AW and CB conceived of the study, and participated in its design and coordination and helped to draft the manuscript. All authors read and approved the final manuscript.

## Supplementary Material

Additional file 1 MoPrP105-132 is found in lipid rafts in primary neurons. Primary neurons were treated with rhodamine-labeled MoPrP105-132 (A) or control scrambled peptide (B) and Alexa Fluor 488 labeled CTxB as described in Figure 1. Click here for file

Additional file 2 MoPrP105-132 avoids early endosomes and lysosomes in primary neurons. Primary neurons were treated with biotin-labeled MoPrP105-132 and transferrin positive early endosomes (A) and LAMP-1 positive lysosomes (B) identified as described in Figure 2 Click here for file

Additional file 3 Neurotoxicity of MoPrP105-132 and inhibition by Squalestatin. NB4 neuroblastoma cells were plated at 3 × 10^4 ^cells/well into 96-well microtiter plates and allowed to adhere overnight. The following day, cells were treated with (A) either MoPrP105-132 or rhodamine labeled MoPrP105-132 and cell viability determined 24 h later. (B) Alternatively cells were treated for 24 h with 100 nM squalestatin or carrier (DMSO) prior to addition of MoPrP105-132 and incubation for 24 h. Cell viability was determined using the MTT method Optical density was measured at 595 nm, and results calculated by reference to untreated cells. Click here for file

Additional file 4 Raw images of the data from manuscript Figure 1A. NB4 neuroblastoma cells were incubated for 30 minutes with 30 μM rhodamine-labeled MoPrP105-132 and lipid rafts revealed by CTxB-Alexa Fluor 488 staining as described in Materials and Methods. Serial sections of the stained cells were taken at 0.2 μm intervals through the cell centre. OpenLab software was used to perform digital deconvolution to remove out of focus light from images and also to determine co-localisation. Click here for file
