# Supplementary figures and images for: Squalestatin alters the intracellular trafficking of a neurotoxic prion peptide
Source: BMC Neurosci. 2007 Nov 22;8:99. doi: 10.1186/1471-2202-8-99 (PMC2131757; doi:10.1186/1471-2202-8-99)

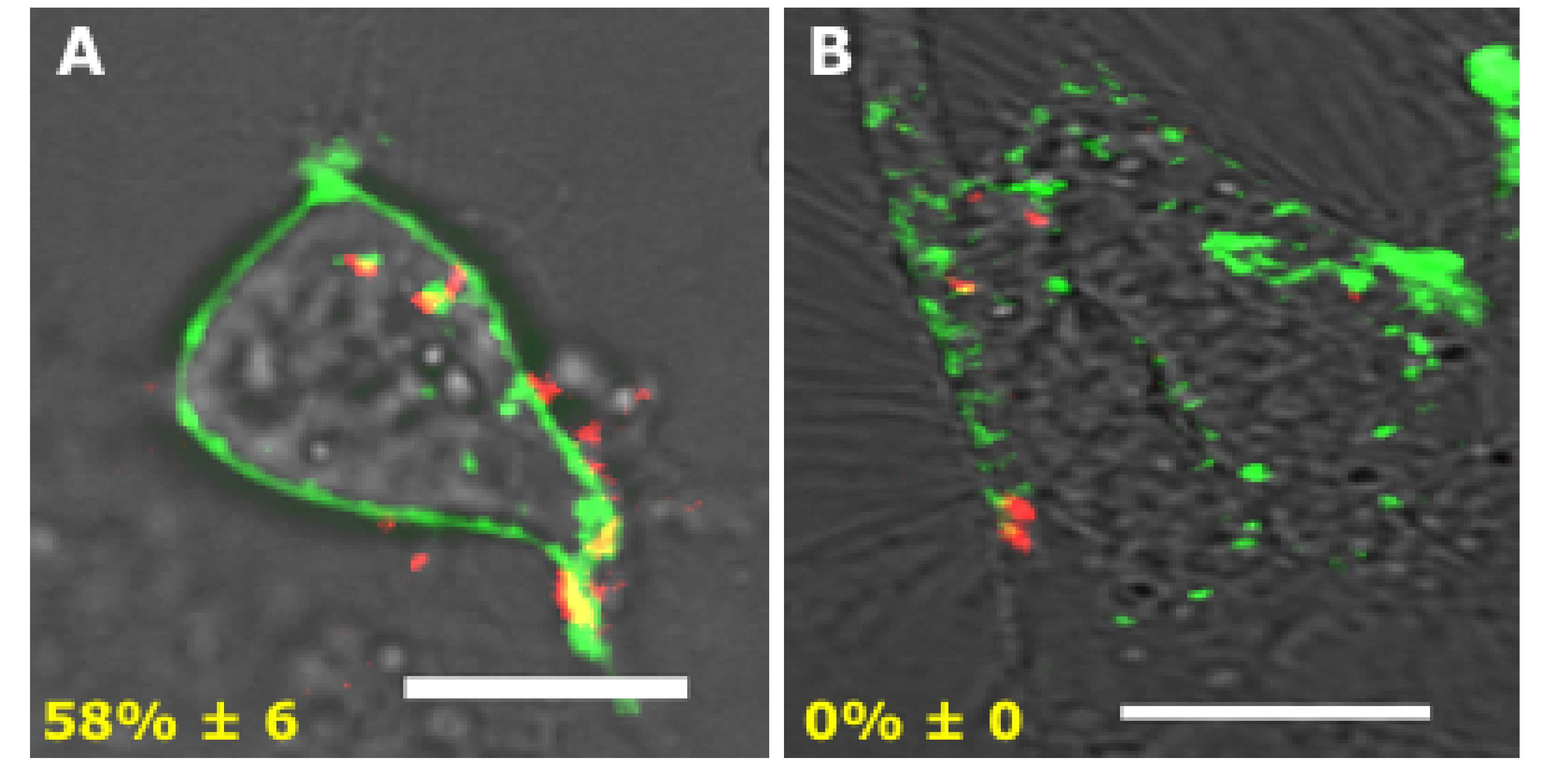

Supplement: Additional file 1 — MoPrP105-132 is found in lipid rafts in primary neurons. Primary neurons were treated with rhodamine-labeled MoPrP105-132 (A) or control scrambled peptide (B) and Alexa Fluor 488 labeled CTxB as described in Figure 1. [file 1471-2202-8-99-S1.jpeg]

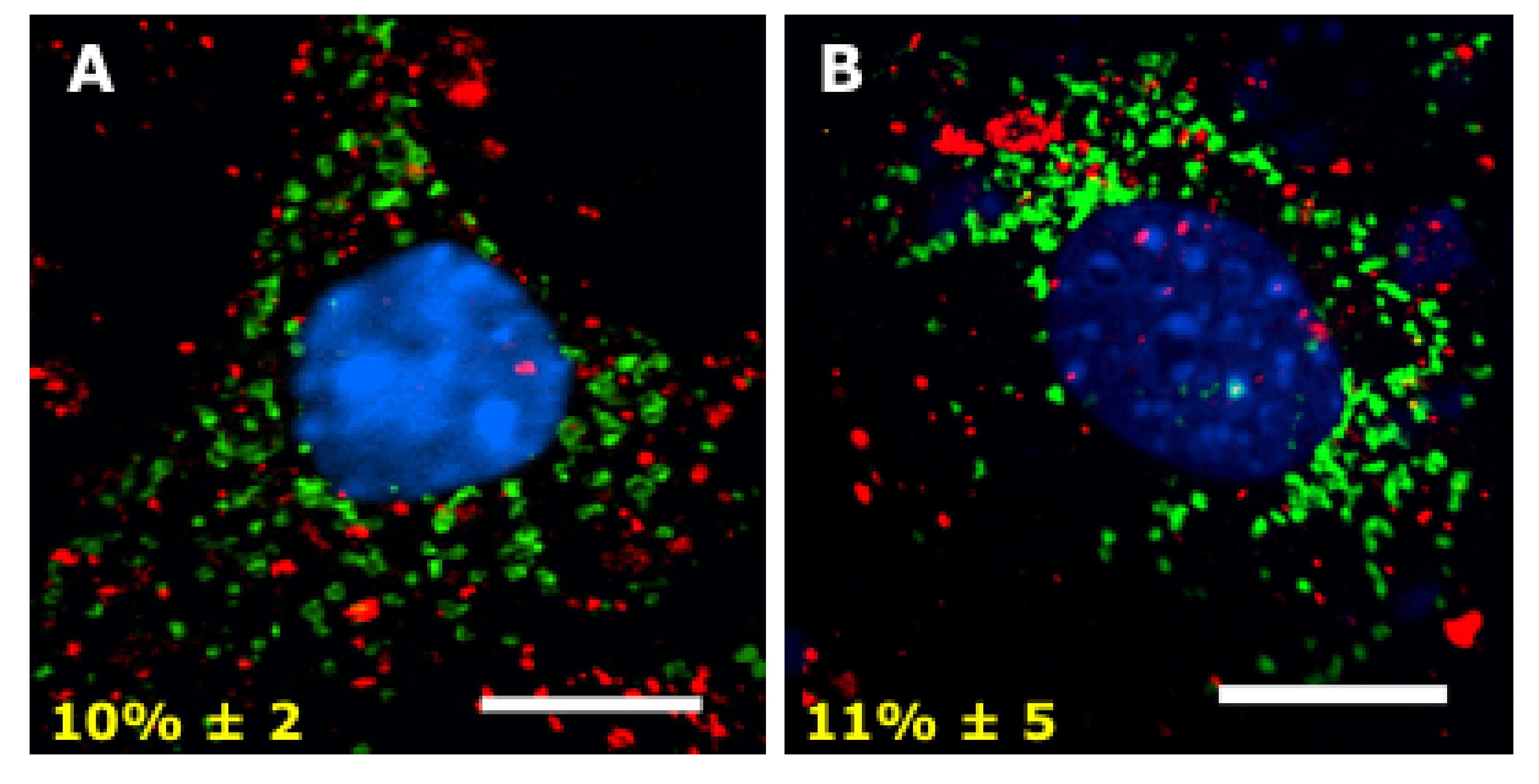

Supplement: Additional file 2 — MoPrP105-132 avoids early endosomes and lysosomes in primary neurons. Primary neurons were treated with biotin-labeled MoPrP105-132 and transferrin positive early endosomes (A) and LAMP-1 positive lysosomes (B) identified as described in Figure 2 [file 1471-2202-8-99-S2.jpeg]

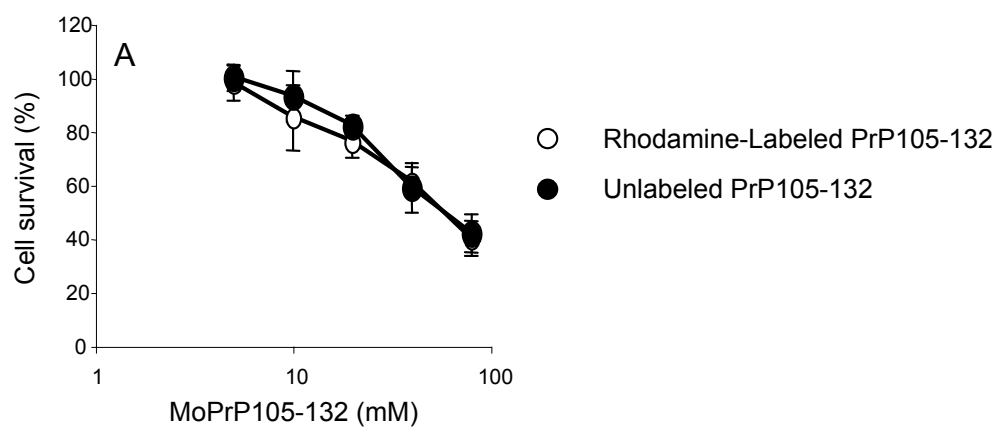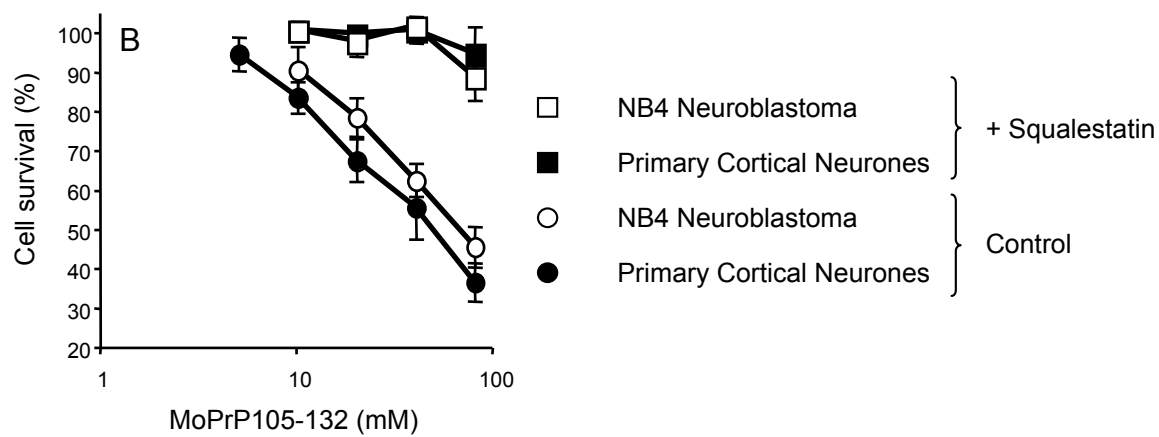

Supplement: Additional file 3 — Neurotoxicity of MoPrP105-132 and inhibition by Squalestatin. NB4 neuroblastoma cells were plated at 3 × 10^4^ cells/well into 96-well microtiter plates and allowed to adhere overnight. The following day, cells were treated with (A) either MoPrP105-132 or rhodamine labeled MoPrP105-132 and cell viability determined 24 h later. (B) Alternatively cells were treated for 24 h with 100 nM squalestatin or carrier (DMSO) prior to addition of MoPrP105-132 and incubation for 24 h. Cell viability was determined using the MTT method Optical density was measured at 595 nm, and results calculated by reference to untreated cells. [file 1471-2202-8-99-S3.pdf]

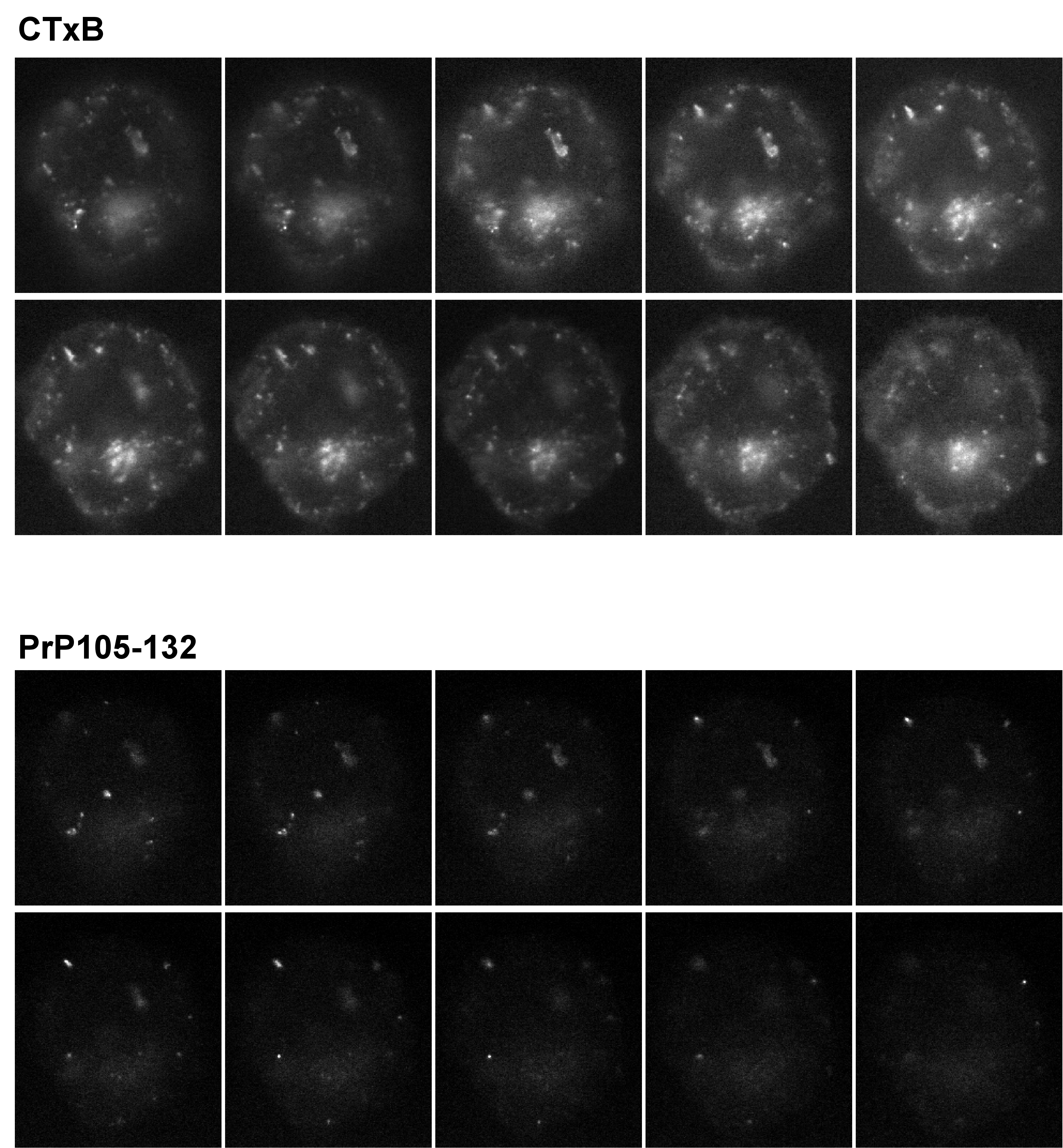

Supplement: Additional file 4 — Raw images of the data from manuscript Figure 1A. NB4 neuroblastoma cells were incubated for 30 minutes with 30 μM rhodamine-labeled MoPrP105-132 and lipid rafts revealed by CTxB-Alexa Fluor 488 staining as described in Materials and Methods. Serial sections of the stained cells were taken at 0.2 μm intervals through the cell centre. OpenLab software was used to perform digital deconvolution to remove out of focus light from images and also to determine co-localisation. [file 1471-2202-8-99-S4.jpeg]
